# Sugar transport in thermophiles: Bridging lignocellulose deconstruction and bioconversion

**DOI:** 10.1093/jimb/kuae020

**Published:** 2024-06-12

**Authors:** Hansen Tjo, Jonathan M Conway

**Affiliations:** Department of Chemical & Biological Engineering, Princeton University, Princeton, NJ 08544, USA; Department of Chemical & Biological Engineering, Princeton University, Princeton, NJ 08544, USA; Omenn-Darling Bioengineering Institute, Princeton University, Princeton, NJ 08544, USA; Andlinger Center for Energy and the Environment, Princeton University, Princeton, NJ 08544, USA; High Meadows Environmental Institute, Princeton University, Princeton, NJ 08544, USA

**Keywords:** Sugar transport, Thermophile, ABC transporter, Lignocellulose, Metabolic engineering

## Abstract

Biomass degrading thermophiles play an indispensable role in building lignocellulose-based supply chains. They operate at high temperatures to improve process efficiencies and minimize mesophilic contamination, can overcome lignocellulose recalcitrance through their native carbohydrate-active enzyme (CAZyme) inventory, and can utilize a wide range of sugar substrates. However, sugar transport in thermophiles is poorly understood and investigated, as compared to enzymatic lignocellulose deconstruction and metabolic conversion of sugars to value-added chemicals. Here, we review the general modes of sugar transport in thermophilic bacteria and archaea, covering the structural, molecular, and biophysical basis of their high-affinity sugar uptake. We also discuss recent genetic studies on sugar transporter function. With this understanding of sugar transport, we discuss strategies for how sugar transport can be engineered in thermophiles, with the potential to enhance the conversion of lignocellulosic biomass into renewable products.

**One-Sentence Summary:**

Sugar transport is the understudied link between extracellular biomass deconstruction and intracellular sugar metabolism in thermophilic lignocellulose bioprocessing.

## Graphical Abstract

**Graphical Abstract:**
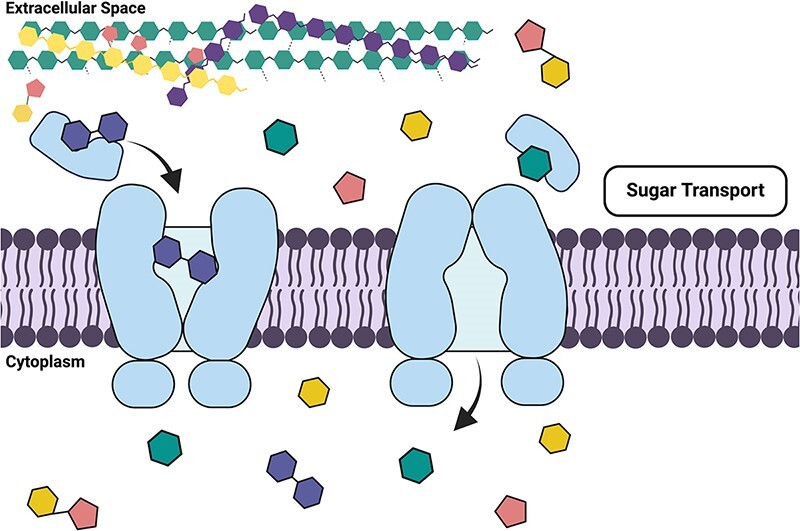

## Introduction

The global pivot toward bio-based supply chains promises to reshape food and energy landscapes, economic development priorities, and national security policy (Lynd, 2017; Jeffery, 2024). In 2022, U.S. President Biden signed Executive Order 14081 (https://www.federalregister.gov/documents/2023/04/27/2023-08841/executive-order-14081-advancing-biotechnology-and-biomanufacturing-innovation-for-a-sustainable-safe), a landmark directive supercharging domestic biomanufacturing through interagency action (The White House, 2022; Gillespie, 2024; Jeffery, 2024). Meanwhile, industry analysis projects a U.S. bioeconomy valued at $4 trillion by 2040 (Gillespie, 2024). Strategic industrial sectors that cannot undergo electrification, such as aviation and maritime shipping, will require renewable carbon neutral fuel due to the insufficient energy densities of electric batteries and hydrogen gas (Fulton et al., 2015; Lynd, 2017; Hannula & Reiner, 2019). Chemical commodities, such as organic acids and alcohols, for the manufacturing of everyday items from plastics, clothes, and composites will also require more sustainable, bio-based production lines (Francois et al., 2020). Yet, further advancement in lignocellulose conversion technologies is critical to meet this growing demand for biomass-derived products, chemicals, and fuels (Hodgson et al., 2002).

Lignocellulose recalcitrance has been an enduring challenge to create a renewable bioeconomy. Although many industrially relevant microorganisms are capable of metabolizing the smallest constitutive elements of lignocellulose—glucose, xylose, and arabinose—they suffer from either an inability to simultaneously uptake and utilize both hexose and pentose sugars, such as baker's yeast (*Saccharomyces cerevisiae*), or the inability to chemically hydrolyze complex cellulose and hemicellulose into smaller oligosaccharides and monosaccharides (*Escherichia coli*) (Hahn-Hägerdal et al., 2006). These limitations have led to the deployment of thermophilic microbes for lignocellulosic fermentation due to several distinct advantages (Blumer-Schuette et al., 2014; Zeldes et al., 2015; Crosby et al., 2019; Jiang et al., 2022; Vavitsas et al., 2022). Firstly, their growth and metabolic conditions are optimized at elevated temperatures, enabling increased biomass solubility, enhanced diffusion coefficients that reduce molecular transport bottle-necks, and minimization of contamination risks by undesired mesophilic microbes (Akinosho et al., 2014; Chung et al., 2014; Zeldes et al., 2015; Crosby et al., 2019; Jiang et al., 2022). Many thermophiles are naturally cellulolytic, armed with native carbohydrate-active enzymes (CAZymes) and glycoside hydrolases (GHs) capable of hydrolyzing cellulose and/or hemicellulose (Blumer-Schuette et al., 2014; Straub et al., 2018; Crosby et al., 2019). This also allows thermophiles to perform consolidated bioprocessing: simultaneous enzymatic breakdown and direct conversion of lignocellulose to products, improving process efficiency (Cha et al., 2013; Akinosho et al., 2014; Chung et al., 2014). Several reviews over the past decade have effectively summarized the advantages of thermophiles as metabolic engineering hosts (Blumer-Schuette et al., 2008, 2014; Zeldes et al., 2015; Crosby et al., 2019; Bing et al., 2021; Jiang et al., 2022). Many thermophiles can grow on a wide range of carbohydrate sources, from long-chained oligosaccharides to disaccharides, pentose sugars to hexose sugars, enabling them to utilize the full diversity of substrates available following lignocellulose deconstruction (Blumer-Schuette et al., 2008; Brunecky et al., 2013; Cha et al., 2013; Straub et al., 2019). Finally, while model mesophiles such as *E. coli* or *S. cerevisiae* have historically been preferred as metabolic engineering workhorses for their superior genetic tractability, the gap has narrowed in recent years as an increasing number of selection and counter-selection markers, libraries of promoters, and even CRISPR-based genome-modification tools for non-model thermophiles have been introduced (Blumer-Schuette et al., 2008; Zeldes et al., 2015; Crosby et al., 2019). And yet, we still know remarkably little about how sugars are transported in biotechnologically significant thermophiles.

**Fig. 1.**
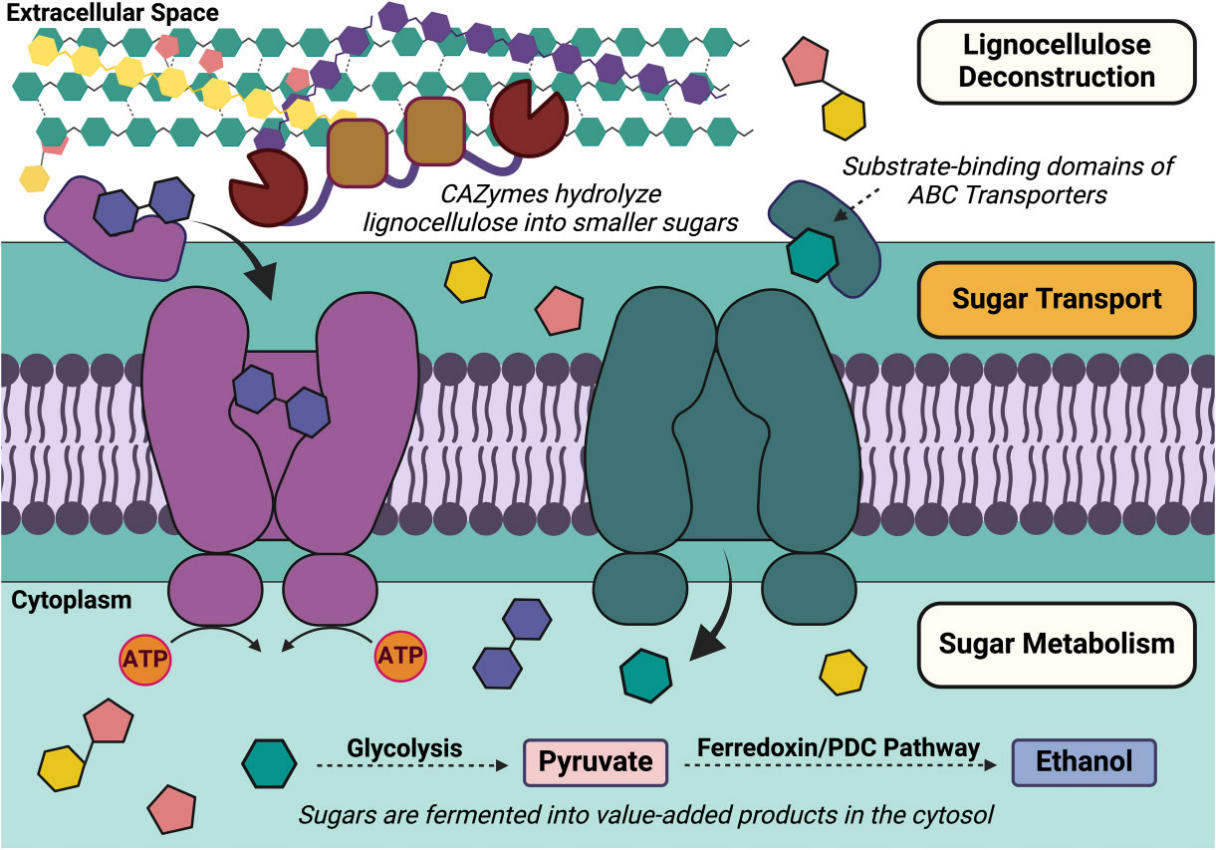
Sugar transport is the understudied link between lignocellulose deconstruction and sugar metabolism in lignocellulose bioprocessing. In thermophiles, sugar transport of both oligosaccharides and monosaccharides is primarily mediated by ATP-Binding Cassette (ABC) sugar transporters powered by ATP hydrolysis. Transported sugars are then converted into value-added products via various metabolic pathways within the cytosol. (Created using Biorender)

In lignocellulose bioprocessing literature, there has been extensive research on both biomass deconstruction through carbohydrate-active enzymes (Brunecky et al., 2013, 2017; Conway et al., 2018, 2018; Yi-Heng & Lynd, 2005) and the engineering of thermophilic metabolism through genetic and pathway manipulation for chemical production (Chandrakant & Bisaria, 1998; Cha et al., 2013; Chung et al., 2014, 2015; Fig. 1). However, the critical interface of these well-explored domains—transmembrane sugar transport, bringing extracellular sugars into the cell for metabolism—has not been as thoroughly examined. Moreover, many industrially relevant thermophiles benefit from a robust metabolism that enables simultaneous pentose and hexose sugar utilization. As such, the mechanisms governing the entry of deconstructed sugar molecules into the cell represent an untapped opportunity for metabolic engineering.

In 2010, Young et al. introduced “panmetabolic engineering,” a novel approach integrating classical biochemical characterization, evolutionary engineering of sugar transporters, and manipulation of sugar utilization pathways in *S. cerevisiae* to enhance lignocellulose conversion (E. Young et al., 2010). This approach challenged a prevailing consensus that internal cellular pathways, rather than molecular substrate transport, are the limiting factors in achieving desired metabolic products (E. Young et al., 2010). Contrary to treating transporter engineering and internal metabolic pathway manipulation as separate, their interdependence underscores the need to optimize both stages simultaneously for significant enhancements in lignocellulose conversion efficacy. However, multiple cellulolytic thermophiles do not share a principal constraint in *S. cerevisiae* whereby a heterologous isomerase or oxidoreductase pathway must be incorporated for pentose sugar utilization (*Clostridium thermocellum* is a noteworthy exception) (E. Young et al., 2010). Conversely, the utility of many cellulolytic thermophiles, for example, *Caldicellulosiruptor, Anaerocellum*, and *Thermoanaerobacterium* species, is that they already possess native pentose and hexose sugar metabolic pathways, while other thermophiles such as *Pyrococcus, Clostridium, Thermotoga*, and *Thermococcus* can utilize oligosaccharides of various lengths as independent carbohydrate sources (Vanfossen et al., 2009; Stincone et al., 2015; Scott et al., 2019; Watanabe et al., 2019; Koendjbiharie et al., 2020).

There have been excellent, albeit dated, reviews on the mechanisms of sugar transporter systems in hyperthermophilic archaea (de Vos et al., 1998; Koning et al., 2002; Davidson & Chen, 2004; Davidson et al., 2008). In many thermophiles, the primary mode of sugar transport relies on the ATP-Binding Cassette (ABC) active transport system. The superfamily of ABC sugar transporters are divided into the carbohydrate uptake transporter (CUT) 1 and 2 families, and are collectively responsible for the transport of the broad range of monosaccharides and oligosaccharides available for microbial fermentation following lignocellulose degradation (Schneider, 2001; Vanfossen et al., 2009). Moreover, the phosphoenolpyruvate-dependent phosphotransferase (PTS) sugar transporter system typically mediates carbon catabolite repression in mesophilic hosts (Warner & Lolkema, 2003; Deutscher et al., 2006, 2014; Görke & Stülke, 2008); however, there have been comparatively few studies on its influence in thermophiles (Saier & Reizer, 1994; Lengeler & Jahreis, 2009; Pickl et al., 2012; McCoy et al., 2015; Bidart et al., 2023).

In this article, we survey sugar transport in long studied archaea and bacteria to lignocellulose degrading and industrially relevant *Anaerocellum, Caldicellulosiruptor, and Clostridium* species. Though we will primarily focus on ABC sugar transport given its significance in many thermophiles, we will also provide an overview of the PTS with contemporary engineering examples. We next consider opportunities for sugar transporter engineering using examples and recent developments from both mesophiles and thermophiles. We end by recasting sugar transport as a synthetic biology control strategy for engineered chemical production in thermophilic microorganisms.

## Sugar Transport in Thermophiles

Sugar transport in thermophiles work similarly as in mesophiles, primarily involving either facilitated diffusion or active transport. The latter process depends on either phosphoenolpyruvate (PEP), as is the case for the phosphotransferase system (PTS), or ATP, which fuel ABC transporters.

Facilitated diffusion uses concentration gradients to translocate sugars across the cell membrane, aided by sugar permease complexes or the parallel diffusion of ions in symporters, for example, the well-characterized proton and arabinose symporter AraE (Kundig et al., 1964; Roseman, 1969; Maiden et al., 1988; Saier & Reizer, 1994; Deutscher et al., 2006, 2014; Lengeler & Jahreis, 2009). Facilitated diffusion is typically more common for smaller sugars such as monosaccharides and disaccharides due to the restrictive binding site and conformation of the sugar permeases involved (Deutscher et al., 2006, 2014; McCoy et al., 2015).

**Fig. 2.**
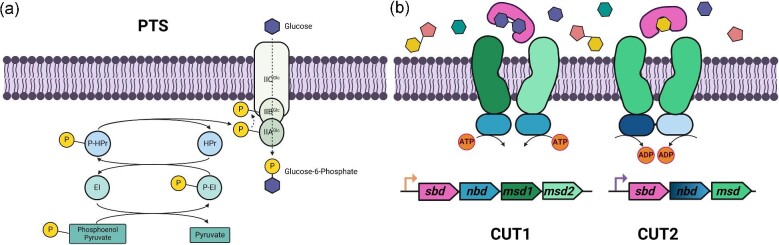
Microbial sugar transport is mediated by both the phosphoenolpyruvate PhosphoTransferase System (PTS) and a superfamily of ATP-Binding Cassette (ABC) Transporters. (**a**) Schematic of the glucose PTS system in *E. coli* whereby the enzyme complex IIA and IIB are fused to the transmembrane domain IIC. Phospho-carriers EI and HPr carry phosphate from PEP to the EII complex. EII phosphorylates the sugar as it is transported. (**b**) The superfamily of ABC sugar transporters can be divided into CUT1 and CUT2 families. CUT1 transporter operons typically contain four gene products: a single substrate-binding domain (sbd), two distinct hydrophobic polypeptides as membrane-spanning domains (msd1 and msd2), which interact with two copies of the same nucleotide-binding domain (nbd). On the other hand, CUT2 transporters contain three gene products: one substrate-binding domain (sbd), one membrane-spanning domain (msd) expressed as a homodimer, along with a distinct nucleotide-binding domain (nbd). This nucleotide-binding domain is characterized by a fusion of two sets of protein sequence motifs essential for ATP binding: Walker A and Walker B. Despite their structural differences, both CUT1 and CUT2 transporters require the hydrolysis of two ATP molecules into ADP for the translocation of one sugar molecule across the membrane. (Created using Biorender)

Like facilitated diffusion, PEP-dependent PTS transporters preferentially target monosaccharides and disaccharides over longer oligosaccharides (Patni & Alexander, 1971; Pickl et al., 2012). As a specialized form of active transport, PTS transporters uniquely couple chemical modification of sugars with their translocation, whereby the imported sugar is phosphorylated as it crosses the membrane (Fig. 2a; McCoy et al., 2015). This phosphoryl group originates from PEP, which first donates its phosphoryl group to enzyme I (EI), followed by sequential phosphate transfers through EI to Histidine-containing phosphocarrier (HPr), then finally enzymes EIIA and EIIB. EIIB typically forms a complex with the transmembrane permease, EIIC, to phosphorylate the sugar as it is internalized. The enzymes EIIA and EIIB can also be sugar specific, with one genome potentially harboring multiple variants of EIIA and EIIB to enable transport of multiple unique sugars using shared EI and HPr components.

PTS transporters have been studied in two thermophiles: *Parageobacillus thermoglucosidasius* and *Thermoanaerobacter saccharolyticum* (Tsakraklides et al., 2012; Bidart et al., 2023). In *P. thermoglucosidasius*, deletions of PTS components confirmed PTS-dependent translocation of common lignocellulosic sugars (cellobiose, fructose, glucose, mannose, sucrose, and trehalose) (Bidart et al., 2023). Deletion of Enzyme I in a ∆*ptsI* strain eliminated growth on these sugars, while normal growth persisted on maltose and xylose thereby indicating alternative transport mechanisms (Bidart et al., 2023). Although the ∆*ptsI* strain maintained xylose growth, subsequent deletion of xylose-specific EIIB abolished growth on xylose, revealing the continued dependence on facilitated diffusion catalyzed by the xylose-specific enzyme II despite the absence of phosphorylation (Bidart et al., 2023). The relationship of PTS with carbon catabolite repression was also explored in *T. saccharolyticum* (Tsakraklides et al., 2012). Despite being able to grow on both hexose and pentose sugars, *T. saccharolyticum* exhibits carbon catabolite repression when grown in a mixture of sugars (Lin et al., 2011; Tsakraklides et al., 2012). Previous literature has identified the presence of *T. saccharolyticum* PTS proteins orthologous to PTS components in *B. subtilis* (K. D. Singh et al., 2008). A strain with a His15Asp mutation in HPr exhibited altered sugar preferences, showing improved growth on arabinose and galactose but reduced glucose and mannose consumption compared to the wild-type (Tsakraklides et al., 2012). Xylose consumption remained largely unaffected likely due to putative ABC transporters for xylose transport, based on genome annotation (Tsakraklides et al., 2012).

Besides the PTS, ABC sugar transporters play a crucial role in sugar uptake. Shown in Fig. 2b, ABC transporters comprise multiple domains: an extra-cytoplasmic substrate-binding domain responsible for sugar-specific binding, a membrane spanning domain, and a nucleotide-binding domain (ATPase) (Ehrmann et al., 1998; Schneider & Hunke, 1998; Schneider, 2001; Hollenstein et al., 2007; Holland, 2019). While membrane-spanning and substrate-binding domains typically sit adjacent to one another in the same genetic locus, ATP-binding domains are sometimes located elsewhere in the genome due to their multifunctionality (Rodionov et al., 2021). As the naming suggests, unidirectional sugar transport is energetically coupled to ATP hydrolysis, typically at a ratio of two ATP molecules to one sugar molecule (Saurin et al., 1999).

ABC sugar transporters can be divided between CUT families 1 and 2 (Schneider, 2001). Structurally, both CUT1 and CUT2 families contain transmembrane and nucleotide-binding (ATPase) domains. CUT1 transporters are translated from four gene products: the extracellular substrate-binding protein, the ATPase domain, and two separate integral membrane polypeptides (Schneider, 2001). These four gene products are organized into a complex, with a membrane-spanning domain containing two separate polypeptides bound to two copies of the same ATPase domain (Schneider & Hunke, 1998; Schneider, 2001). While members of the CUT2 family also contain extracellular and ATPase domains, they contain one less gene product compared to CUT1 family members due to the transmembrane domain itself being a homodimer (Schneider, 2001). The ATPase in CUT2 family members is a fusion protein of two sets of Walker A and Walker B protein sequence motifs essential for ATP binding, in contrast to the homodimeric nature of CUT1 nucleotide-binding domains (Schneider & Hunke, 1998; Schneider, 2001). Finally, extracellular substrate-binding proteins, which are highly ligand specific, dictate the sugars imported by an ABC transporter (Berntsson et al., 2010; Bosdriesz et al., 2015; Scheepers et al., 2016). Substrate recognition is also divided along CUT1 and CUT2 family lines: the CUT2 family almost exclusively transports monosaccharides, whereas the CUT1 family transports a wider variety of substrates ranging from oligosaccharides to glycerol-phosphates (Schneider, 2001).

The past decade has shown that many thermophiles of biotechnological importance possess multiple CUT1 and CUT2 ABC transporters for monosaccharide and oligosaccharide uptake: *Thermus thermophilus, Thermotoga maritima, Pyrococcus furiosus, Caldicellulosiruptor saccharolyticus, Anaerocellum bescii* (D. A. Rodionov et al., 2013), (VanFossen et al., 2009; Stincone et al., 2015), (D. A. Rodionov et al., 2021), (Chandravanshi et al., 2019). The ABC cellodextrin and glucose transporters in *C. thermocellum* have also garnered significant attention (Nataf et al., 2009; Yan et al., 2022). Between both CUT1 and CUT2 ABC transporters, thermophiles are well-equipped to uptake the diverse array of sugars in their native environments, which tend to be limiting in growth substrate (Koning et al., 2002; Bosdriesz et al., 2015). Possessing multiple types of ABC sugar transporters likely confers advantages with efficient scavenging.

And, thermophilic ABC sugar transport exhibits interesting physiological features—in part due to their carbohydrate limiting native environments. For example, in *T. maritima*, maltose uptake is mediated by three separate *malEGFK* operons, each containing isoforms of maltodextrin-binding proteins (TmMBP1, TmMBP2, TmMBP3) that display distinct binding properties. While TmMBP1 and TmMBP2 exhibit a sequence similarity of over 90% and tightly bind to longer maltodextrins such as maltotriose and maltotetraose, TmMBP3 only preferentially binds maltose (and glucose to a lesser extent) while being 40% identical in sequence. Thus, redundant ABC sugar transporter operons with differential substrate preferences can enable more efficient saccharide scavenging despite the metabolic costs of a larger genome size (Latif et al., 2015; R. Singh et al., 2017). Interestingly, only one *malK* gene is ostensibly responsible for powering all three maltodextrin transporters out of the three putative *malK* genes encoding an ATPase for MalEGFK in *T. maritima*; its deletion was both necessary and sufficient to disrupt maltodextrin transport (R. Singh et al., 2017).

An external abundance of cognate sugars can lead to reduced expression of substrate-binding proteins (Nanavati et al., 2002). At higher extracellular concentrations of sugars, lower concentrations of periplasmic substrate-binding proteins are sufficient in saturating ABC translocators (Bosdriesz et al., 2015). Interestingly, a different form of repression-based control is exhibited by the *P. furiosus* and *Thermococcus litoralis mal* operons. When intracellular levels of maltose and trehalose are too low, TrmB, a sugar-sensing transcriptional repressor, remains bound to the promoter region upstream of the maltose/trehalose ABC operon to inhibit transcription, preventing energy expenditure on transporters unlikely to be saturated consistently (S.-J. Lee et al., 2007a, 2007b, 2008).

## Substrate-Binding Proteins Determine ABC Transporter Specificity

The defining feature of ABC sugar transporters is their extracellular substrate-binding proteins (SBP) that recognize target carbohydrates with high binding affinities. These substrate-binding proteins indicate the sugars that their corresponding translocator assimilates. Moreover, their extracellular nature makes them comparatively easier to heterologously express and purify for *in vitro* characterization than other membrane-bound components of an ABC transporter. Common approaches for studying sugar transport using substrate-binding proteins include structural biology to delineate important amino acid residues coordinating high-affinity interactions, and biophysical measurements to determine the thermodynamics and differential substrate-binding preferences.

**Fig. 3.**
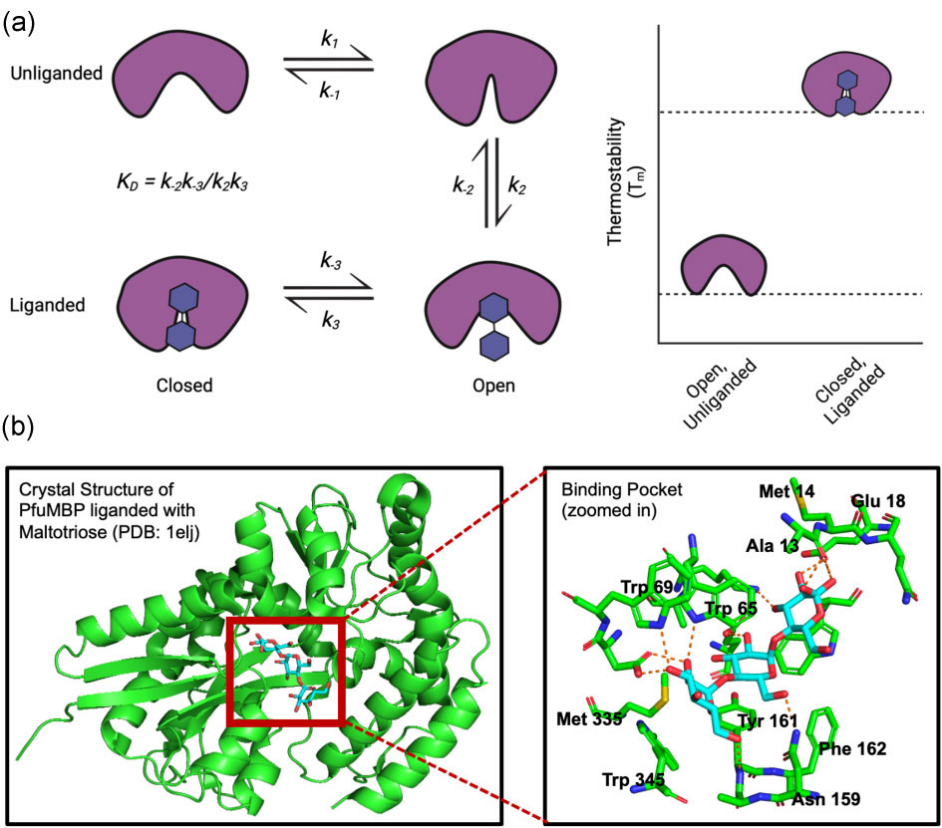
Substrate-binding proteins are conformationally dynamic. (**a**) Substrate-binding proteins sample various conformations that include open, unliganded forms to closed, liganded forms. The presence of target substrates shifts chemical equilibrium towards the closed, liganded conformation, which is both thermodynamically and thermally more stable. (**b**) 3D structure of the liganded *Pyrococcus furiosus* maltodextrin-binding protein (PfuMBP) (PDB: 1elJ) co-crystallized with maltotriose substrate. Zooming into the binding pocket shows maltotriose (cyan, in stereo representation) and hydrogen bonds with residues within a 3.5 Å radius. Carbon atoms are colored green, oxygen atoms are colored red, nitrogen atoms are colored blue, and sulfur atoms are colored yellow. (Created using Biorender)

Genes encoding SBPs are typically, though not exclusively, positioned adjacent to genes expressing the membrane-spanning and ATP-binding domains of tri-modular ABC sugar transporter operons. Structurally, they exhibit a wide range of sizes from 25 kDa to 70 kDa (Berntsson et al., 2010; Scheepers et al., 2016). Despite SBPs generally bearing low sequence homology to one another, they typically exhibit high conservation of a two-domain fold (Fig. 3a). Each of the two structurally conserved domains comprise a central β-sheet containing five β-strands and surrounded by α-helices, and are linked by a hinge region that mediates the transition between open and closed conformations, shown by the example of *P. furiosus* maltose-binding protein (PfuMBP) (Fig. 3b; Berntsson et al., 2010; Scheepers et al., 2016; de Boer et al., 2019). In an open conformation, the SBP is flexible around the hinge, while the presence of a substrate capable of binding induces a closed conformation typically marked by greater thermostability and the substrate positioned at the center of the two domains (Fig. 3a). For example, the binding of maltose to *E. coli* MalE elevates its melting temperature by 8–15°C (Novokhatny & Ingham, 1997; Nickolaus et al., 2020). This SBP mechanism of opening and closing has typically been referred to as the “Venus Fly-trap” mechanism (de Boer et al., 2019). In Gram-positive bacteria, extracellular solute-binding proteins are tethered to the plasma membrane through an N-terminal cysteine residue connected to a diglyceride via thioether linkage, whereas substrate-binding proteins freely float in the periplasm in Gram-negative bacteria (Horlacher et al., 1998; Nataf et al., 2009). Finally, no cofactor or catalyst is known to be necessary to drive conformational change in substrate-binding proteins other than the sufficiently high concentration, typically several orders above the substrate dissociation constant, *K_D_*, of putative substrates in solution with protein (Hülsmann et al., 2000; Jones et al., 2000; Wassenberg et al., 2000; Diez et al., 2001).

Maltodextrin-binding proteins, encoded by the gene *malE*, serve as a model for studying ABC sugar transport in thermophiles. Their genes are somewhat highly conserved, with extensive information available on the *E. coli* MalE homolog (Duplay et al., 1984; Spurlino et al., 1991; Sharff et al., 1992). Over 12 maltodextrin-binding proteins from various thermophiles have been identified or characterized to date, exhibiting binding affinity for α-glucans of variable length. These proteins, exemplified in studied thermophiles such as *(1) T. thermophilus, (2) Thermoanaerobacter ethanolicus, (3) P. furiosus, (4) Thermatoga maritima, (5) T. litoralis, and (6) Thermoanaerobacter thermosulfurigenes*, demonstrate unique combinations of hydrogen bonds and van der Waals forces in substrate binding (Herrmann et al., 1996; Sahm et al., 1996; Horlacher et al., 1998; Hülsmann et al., 2000; Jones et al., 2000; Wassenberg et al., 2000; Diez et al., 2001; Greller et al., 2001; Koning et al., 2001, 2002; Silva et al., 2005; Table 1). Additionally, horizontal gene transfer of maltodextrin-binding systems has been identified between thermophiles (Koning et al., 2002).

**Table 1.** Characterized thermophilic ABC substrate-binding proteins and their binding affinities with known carbohydrate substrates.

Thermophile	Substrate-Binding Protein	Sugar	*K_D_* (μ*M*)	T (^∘^C)	References
*Clostridium thermocellum*	CbpA	Glucose	240	30	(Nataf et al., 2009; Yan et al., 2022)
		Cellotriose	0.20*	50	
	CbpB	Cellobiose	1.89–2.70	30	
		Cellotriose	0.62–1.17		
		Cellotetraose	0.48–0.56		
		Cellopentaose	0.53–0.67		
	CbpC	Glucose	0.38		
		Cellobiose	0.44		
		Cellotriose	0.49		
		Cellotetraose	0.38		
		Cellopentaose	0.28		
	CbpD	Cellotriose	0.44		
		Cellotetraose	0.43		
		Cellopentaose	0.22		
	Lbp	Laminaribiose	0.13		
*Pyrococcus furiosus*	PfuMBP	Maltose	–	–	(Evdokimov et al., 2001; Koning et al., 2001, 2002)
		Maltotriose	37	15	
	CbtA	Cellobiose	45	60	
		Laminarin	–	–	
*Sulfolobus solfataricus*	GlcS	Glucose	0.43	60	(Albers et al., 1999; Elferink et al., 2001; Lubelska et al., 2006)
		Galactose	–		
		Mannose	–		
	AraS	Arabinose	0.13		
*Thermatoga maritima*	TmMBP	Maltose	∼0.3	80	(Nanavati et al., 2002; Wassenberg et al., 2000)
		Maltotriose	12.6	20	
*Thermoanaerobacter ethanolicus*	MalE1	Maltose	0.27	70	(Jones et al., 2000)
		Trehalose	0.27		
*Thermococcus litoralis*	TMBP	Maltose	0.16	80	(Horlacher et al., 1998; Diez et al., 2001; Greller et al., 2001;)
		Trehalose	0.16		
*Thermus thermophilus*	MalE1	Trehalose	0.10	70	(Silva et al., 2005)
		Maltose	0.062		
		Sucrose	0.40		

Shown in Table 1, we emphasize that tight, high-affinity binding between substrate-binding proteins and their putative substrates is typical as quantified by micromolar-range dissociation constants. This binding occurs when the energy of association exceeds entropic contributions from dissociation, the latter which scales linearly with temperature (Evdokimov et al., 2001). Binding interactions can be enthalpic, such as Van der Waals associations and hydrogen bonds between glucosyl groups, water molecules, and binding pocket residues; as well as entropic, such as the stacking of pyranosyl rings on aromatic side chains (Evdokimov et al., 2001). In the case of *P. furiosus* MBP (PfuMBP), its binding to maltotriose is stabilized by a hydrophobic stack involving phenylalanine and tryptophan residues, along with over 20 hydrogen bonds formed with glucosyl residues and amino acids in the binding pocket (Fig. 3b). The trehalose-TMBP complex is similarly stabilized by hydrogen bonds with nearby amino acids (Silva et al., 2005).

Interestingly, while tighter side-chain packing and smaller cavities are typically associated with greater thermostability, cavity volumes between PfuMBP and *E. coli* MBP are similar (Evdokimov et al., 2001; Sammond et al., 2016). Both PfuMBP and *E. coli* MBP also contain a comparable number of hydrogen bonds. Instead, the advanced thermostability of PfuMBP may stem from its higher density of salt bridges and proline residues, and biasing toward thermolabile residues such as Asn, Gln, Met, and Cys (Evdokimov et al., 2001). This is intriguing from a structural standpoint, as amino acid composition is not generally considered a driver of thermophilic adaptation (Vieille & Zeikus, 2001). However, a higher density of salt bridges—particularly along the active site, which are present in PfuMBP—promotes greater thermal stability at the expense of reduced activity at lower temperatures (Evdokimov et al., 2001; Lam et al., 2011).

The temperature-dependent dynamics of thermophilic substrate-binding proteins is also not extensively understood (Knight et al., 2023). And though substrate-binding proteins are strictly speaking not enzymes, as they do not alter the chemical nature of their substrates, they still exhibit activity (Berntsson et al., 2010). Consistent with experimental data, it is possible to consider that the Eyring profile of a thermophilic enzyme is similar to its mesophilic counterpart except shifted to a higher temperature (Dotas et al., 2020; Knight et al., 2023). Thus, it is expected that the structural features lending thermophilic enzymes their higher thermal stability would render them less active than mesophilic homologs—at mesophilic temperatures (Lam et al., 2011). Evidence of greater substrate promiscuity by thermophilic enzymes could be driven by their access to a broader distribution of conformations at elevated temperatures (Dotas et al., 2020; Knight et al., 2023). Applying this concept to substrate-binding proteins, it is conceivable that the binding dynamics observed at lower temperatures in Table 1 could differ to what would be observed at higher native temperatures for optimal activity. In other words, increased conformational heterogeneity at elevated temperatures could result in distinct substrate preference and binding behavior to those at lower temperatures.

Investigations into MalE have highlighted its conformational and dynamic plasticity, with implications on the general behavior of thermophilic substrate-binding proteins. Based on solution smFRET efficiency histograms of MalE bound to various cognate maltodextrins with glucosyl units two to seven, de Boer et al. showed how MalE sampling across four different conformational states—(1) open unbound, (2) open bound, (3) closed bound, and (4) closed unbound (Fig. 3a)—is dependent on the degree of cognate substrate binding (Berntsson et al., 2010; Scheepers et al., 2016; de Boer et al., 2019). FRET efficiency histograms suggest three bound conformations of decreasing extent of closure from maltose (G2) to maltoheptaose (G7), suggesting a preferential subset of substrates even amongst all those that can induce binding (Berntsson et al., 2010; Oldham et al., 2013; Scheepers et al., 2016; de Boer et al., 2019).

The conformational plasticity of substrate-binding protein closure is linked to sugar transporter regulation (de Boer et al., 2019). Only an SBP in closed bound conformation can allosterically interact with a translocator, though the nature of the interaction depends on conformational matching. For example, substrate-binding proteins with a non-cognate substrate would either only be partially closed such that it is conformationally mismatched with its corresponding translocator, or a conformational match takes place but translocator limitations prevent passage of the substrate. In other words, the kinetics of substrate transport are substrate-dependent, with different rate-determining steps depending on substrate identity (de Boer et al., 2019). The translocator's binding cavity size also limits longer-chained oligosaccharides' capacity for transport despite reversible binding to the substrate-binding protein: for example, *E. coli* MalE binds to malto-octaose but the oligosaccharide is too big for translocation by MalF/G (de Boer et al., 2019). That substrate-specific interactions govern whether or not uptake occurs suggest a proofreading system akin to the activities of DNA polymerase's 3ʹ–5ʹ exonuclease and aminoacyl tRNA synthetases (Shevelev & Hübscher, 2002; Kotik-Kogan et al., 2005). Interestingly, the active site of MalE can also be mutated to bind other disaccharides such as sucrose at the cost of hindering interactions between MalE and the MalF/G translocator (Gould & Shilton, 2010).

Beyond maltodextrin-binding proteins, a recent study by Yan et al. (2022) has revealed new structural and biophysical insights into cellodextrin transport in *C. thermocellum* (Yan et al., 2022). Five ABC sugar transporters—A, B, C, D, L—collectively mediate cellodextrin and laminaribiose uptake (Nataf et al., 2009; F. Yan et al., 2022). Transporter A (*clo1313_1828–30*) is the sole transporter that facilitates glucose uptake, while Transporter B (*clo1313_1194–6*) is the sole transporter facilitating cellobiose uptake. Along with Transporter B, Transporter C (*clo1313_2783–6*) facilitates cellodextrin uptake (Nataf et al., 2009; F. Yan et al., 2022). The structures of each transporter's substrate-binding proteins—CbpA, CbpB, CbpC, CbpD, CbpL—in their unliganded apo conformations, CbpA bound to glucose, and CbpB bound to an array of cellodextrins were solved (F. Yan et al., 2022). All substrate-binding proteins displayed the characteristic ligand-binding site between two-domains connected by a flexible hinge region (F. Yan et al., 2022). The binding affinity of cellodextrin-binding proteins in *C. thermocellum* were previously studied by Nataf et al. in 2009 and revised by Yan et al. in 2022. There is broad agreement that its cellodextrin-binding proteins tightly bind to preferred substrates in the 0.1–1.0 micromolar range (Nataf et al., 2009; F. Yan et al., 2022). However, the exact determination of substrates for CbpA, CbpC, CbpD, and Lbp have divided the two studies. While Nataf et al. observed tight binding of CbpA to cellotriose, Yan et al. identified CbpA as a glucose transporter that solely transporters glucose and not cellodextrins. Moreover, Yan et al. could not verify the substrates for Transporters D and L, with guanosine considered a putative substrate for Lbp, and therefore a second role of Lbp as a nucleotide-binding protein, whereas Nataf et al. (2009) showed evidence for CbpD binding cellodextrins, as well as Lbp binding laminaribiose. (Nataf et al., 2009; F. Yan et al., 2022).

Bioenergetic optimization may also contribute to differential substrate-recognition by ABC sugar transporters (Yi-Heng & Lynd, 2005; Nataf et al., 2009; F. Yan et al., 2022). Table 1 shows that *C. thermocellum* substrate-binding proteins CbpB, CbpC, and CbpD preferentially bind longer chained cellodextrins over cellobiose based on either lower dissociation constants or an inability to bind cellobiose. As the cost of two ATP molecules remains fixed for the transport of a sugar molecule irrespective of size, *C. thermocellum* is incentivized to consume longer cellodextrins that are comparatively richer sources of ATP than cellobiose following fermentation. Hence, longer cellodextrins are more metabolically efficient. These results are consistent with Zhang & Lynd's observations that *C. thermocellum* preferentially consumes higher-order cellodextrins (>G4) over cellobiose (G2) to maximize ATP generation, a behavior complemented by a native hydrolase mechanism that degrade celluloses into cellodextrin products with an average degree-of-polymerization of “4” (Yi-Heng & Lynd, 2005; Y.-H. P. Zhang & Lynd, 2005; Nataf et al., 2010).

## Molecular Engineering of Sugar Transport

Genes encoding ABC sugar transporters with known substrates can be strategically refactored across microorganisms to introduce new sugar uptake functionalities. Alternatively, existing ABC sugar transporters can be deleted to eliminate uptake of certain sugars altogether, yielding strains capable of orthogonal sugar transport. These approaches can be combined with other metabolic engineering strategies from pathway manipulation to space-time control. Since most microorganisms require carbohydrates for central metabolism, regulating entry points for different sugars facilitates selection of specific strains for desired biological products and co-culture behavior. A deeper knowledge of the principles undergirding sugar transport will pave multiplying opportunities for engineering microbial factories.

**Fig. 4.**
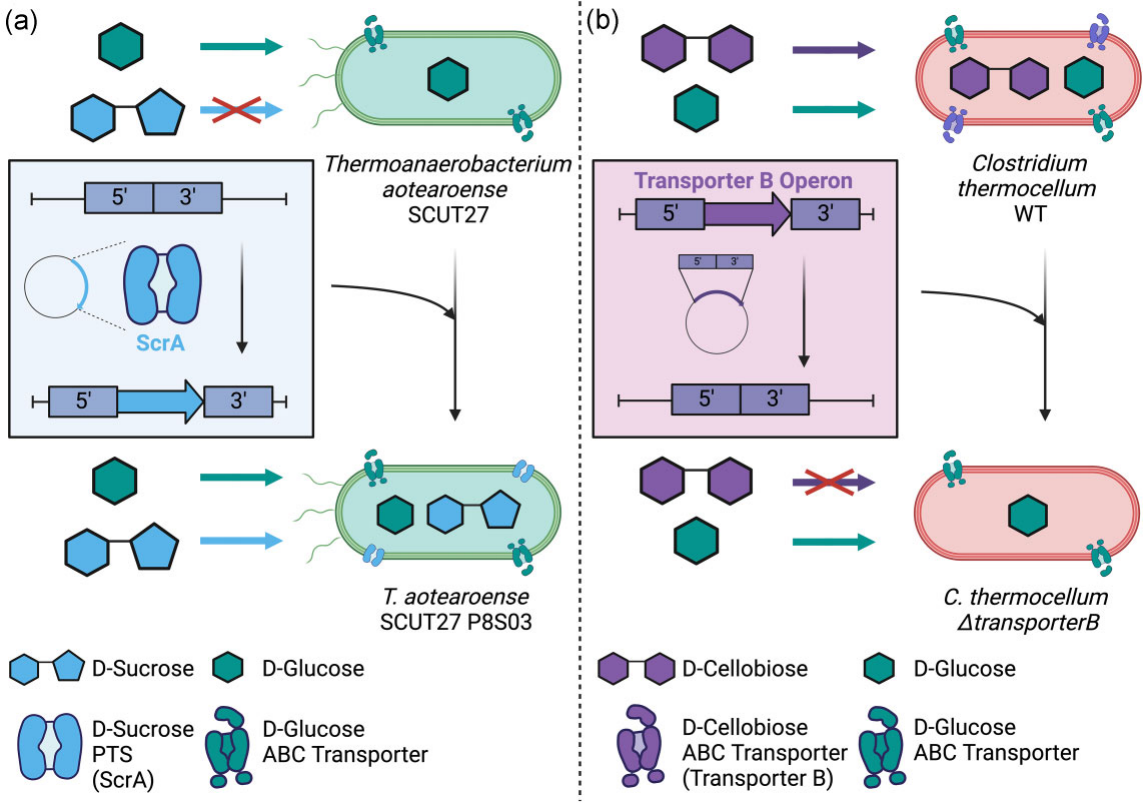
Genetic manipulation of sugar transporters in thermophiles. (**a**) Incorporation of a D-sucrose transporter gene (*scrA*) in *Thermoanaerobacterium aotearoense* SCUT27 to facilitate D-sucrose transport and utilization. (**b**) Deletion of ABC D-cellobiose transporter B in *C. thermocellum* to inhibit cellobiose uptake. (Created using Biorender)

Recent studies have demonstrated successful integration of heterologous sugar transporters in thermophilic workhorses. Shown in Fig. 4a, Dai et al. (2023) engineered *Thermoanaerobacterium aotearoense* SCUT27 to uptake and metabolize sucrose from sugarcane bagasse, through genomic integration of genes encoding for a sucrose PTS system, a sucrose permease system, and a sucrose metabolism system from *Thermoanaerobacterium thermosaccharolyticum* (Dai et al., 2023). The resulting strain was able to utilize sucrose through PTS-mediated sucrose phosphorylation into sucrose-6-phosphate, followed by cytoplasmic catabolism into glucose and fructose. Another example that focused on improving sugar utilization focused on enabling pentose sugar co-fermentation in *C. thermocellum* (Xiong et al., 2018). While wild-type *C. thermocellum* can putatively transport xylose, it cannot effectively grow on pentose sugars (Tafur Rangel et al., 2020). However, the addition of genes encoding the *xylAB* pathway from *T. ethanoliocus* enabled an engineered strain of *C. thermocellum* to co-ferment xylose (including xylo-oligosaccharides) and native cellodextrin substrates (Xiong et al., 2018). Moreover, simultaneous growth on xylose and cellobiose demonstrated an absence of carbon catabolite repression in *C. thermocellum* (Xiong et al., 2018).

Disabling uptake of a native carbon source can also pave strain engineering applications. This is typically achieved through genetic deletion of native sugar transporters, causing a deficiency in corresponding substrate uptake that reduces growth. For example, Fig. 4b illustrates disrupted cellobiose transport in a *C. thermocellum* deletion strain (∆*clo1313_1194–*1196) (Yan et al., 2022). Additionally, ABC sugar transport can also be disrupted by deletion of a corresponding ATP-binding domain. In *A. bescii*, deletion of the ATPase (*msmK*) limited growth on all oligosaccharide carbon sources (Rodionov et al., 2021). This demonstrated that in *A. bescii* one ATP-binding domain appears to be shared by all CUT1 family ABC sugar transporters. These thermophilic strains with disabled sugar uptake are especially useful in co-culture settings, whereby different strains feed on individualized sugars and thereby circumvent carbon source competition.

**Fig. 5.**
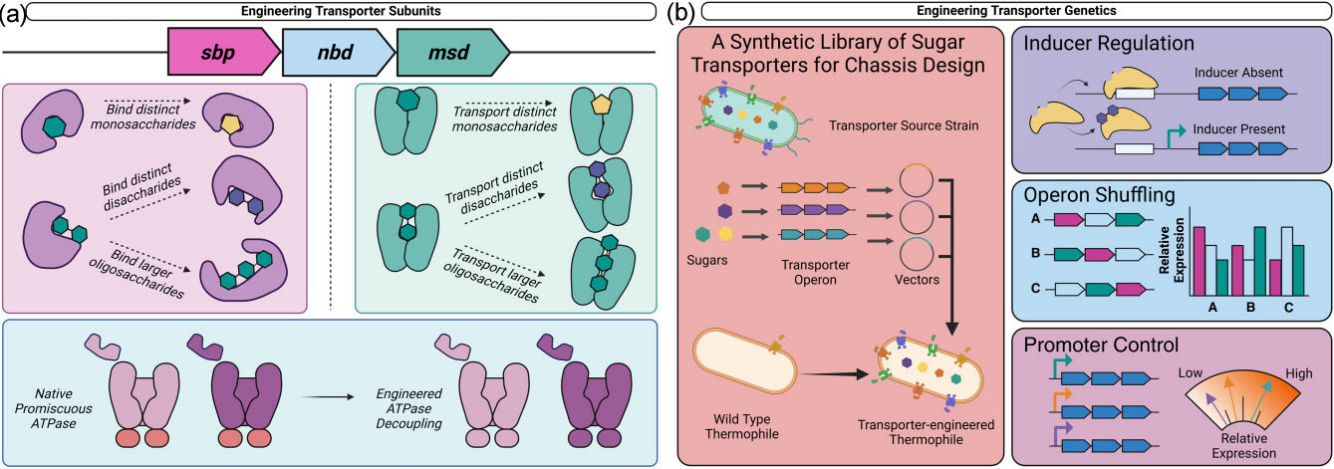
Protein domain and genetic approaches to sugar transporter engineering. (**a**) Sugar transporter subunits can be individually mutated and engineered for changes in substrate-specificity and function. (**b**) Sugar transporters offer new avenues for a range of genetic engineering approaches, including a library of synthetic sugar transporters for bottom-up chassis design of microbial factories, as well as operon-level control over transporter expression. (Created using Biorender)

Thanks to their plasticity, ABC sugar transporters can be rewired to uptake different sugars (Fig. 5a). Young et al. demonstrated this through transporter cloning of glucose transporters *Candida intermedia* GXS1 and *Scheffersomyces stipitis* XUT3 into *S. cerevisiae*, then subjecting refactored strains to directed evolution, resulting in a 70% increase in growth rate on xylose (E. M. Young et al., 2012). Substrate-binding and membrane-spanning domains were the primary beneficiary of point mutations conferring modified roles. Originally glucose transporters, mutated variants supported growth on xylose in *C. intermedia, S. stipitis*, and *S. cerevisiae*. Curiously, these reprogrammed xylose transporters were still transcriptionally repressed by the presence of glucose, highlighting broader expression schemes that remain active despite local modifications to sugar transporters (E. M. Young et al., 2012, 2014).

In thermophiles, a recent study evolved *T. maritima* to favor glucose transport. Under adaptive laboratory evolution conditions, *T. maritima* showed increasing transport and utilization of glucose over successive generations, despite its stronger preference for di- and oligo-saccharides (Latif et al., 2015). Enhanced glucose uptake was correlated to both the upregulation of its native *GluEFK* glucose transporter system and down-regulation of the *BglEFGKL* β-glucoside transporters, with the expression of substrate-binding proteins being the most heavily reduced in the latter case (Latif et al., 2015). The evolved glucose-feeding strains exhibit far lower expression levels of substrate-binding proteins that do not bind to glucose, a key metabolic cost-saving measure that implicates the role of sugar transport in modulating strain fitness (Latif et al., 2015).

Since the literature already contains multiple ABC sugar transporters with well-characterized substrate specificity (Table 1), the logical progression is to establish a library of well-characterized thermophilic ABC sugar transporter parts (Fig. 5b). Because many existing ABC sugar transporter components characterized in mesophiles are unlikely to be stable at high temperatures, this library would fuel efforts for bottom-up chassis design, facilitating the heterologous expression of these well-characterized components in non-native thermophilic microorganisms (Adams, 2016; Jiang et al., 2022). *C. thermocellum* is an excellent candidate for building this library due to the comprehensive characterization of its cellodextrin transporters (Nataf et al., 2009; Yan et al., 2022). Heterologous sugar transporter addition described above with *Thermoanaerobacterium aotearoense* also supports the notion that a species’ native sugar transporter system can be significantly expanded. Providing alternative entry points for native carbon sources may also improve sugar uptake kinetics. The redundancy observed in thermophilic ABC sugar transporters promotes exciting opportunities for studying paralogs uptaking similar substrates. For example, mapping the binding pocket residues that distinguish maltodextrin preferences across TmMBP1, TmMBP2, TmMBP3 support design rules for engineering substrate-binding proteins to selectively bind a particular sugar molecule.

This burgeoning library of thermophilic, heterologous sugar transporters for bottom-up chassis construction also benefits immensely from manipulation of existing sugar transporters. Mutation of key residues on substrate-binding domains can either constrict or expand the range of sugars for binding and consumption depending on the engineering goal (Fig. 5a). The rewiring of hexose transporters into pentose transporters is highly promising from this standpoint (Young et al., 2012, 2014; Li et al., 2016). Still, it is worth noting that mutagenesis of the translocator appears crucial to thoroughly alter substrate transport: a mutated *E. coli* MalE bound to sucrose failed to interact with the *mal* translocator with the same affinity as native MalE bound to maltose (Gould & Shilton, 2010). For thermophiles with more active PTS roles such as *T. saccharolyticum* or *P. thermoglucosidasius*, it is also possible genetic modifications to the PTS system may be necessary for efficient uptake of non-preferred carbon sources.

Adjusting regulation of ABC transporter operons also presents a straightforward approach to engineering sugar transport. Multiple thermophiles possess a single ATPase subunit powering multiple ABC sugar transporters, e.g. the *T. maritima* MalE system, the *A. bescii* CUT1 systems (Singh et al., 2017; Rodionov et al., 2021). Potentially, these sugar transporter operons can be decoupled from the same ATPase subunit, resulting in new phenotypic modes for sugar transport (Fig. 5a). Though *T. maritima* is known to modulate expression of substrate-binding proteins in response to metabolic conservation and substrate-availability, it is also possible that rearranging an ABC transporter's components within the same operon or controlling them with non-native promoters could bypass this regulatory mechanism. Positioning the membrane-spanning domains closer to the promoter, as the most upstream components of the operon, may increase their expression levels compared to most native ABC transporter operons where membrane-spanning domains are typically positioned furthest downstream. Besides engineered operon rearrangement, the incorporation of alternative constitutive or inducible promoters could also tune expression levels (Fig. 5b).

Sugar-sensing regulators also fulfill an important niche in controlling gene expression in thermophiles (Fig. 5b). For instance, the sugar binding site of TrmB from *P. furiosus* could be customized to respond to different sugars, for example, binding β-glucans like cellobiose instead of α-glucans such as maltose or trehalose. Simultaneously, the DNA-binding motif could be engineered to target a region upstream of an ABC transporter operon responsible for translocating alternative substrates. Inputs of chemical inducers such as cellobiose for the native cellulosome in *C. thermocellum* could also be dynamically controlled.

Although we know of multiple ABC sugar transporters across thermophiles that transport both α-glucans and β-glucans, as shown in Table 1, comparatively less is known about transporters that uptake more structurally diverse sugars from lignocellulose such as xylo-oligosaccharides and arabinoxylans. Characterization of such transporters, and their incorporation into thermophiles like *C. thermocellum* that cannot otherwise utilize pentose sugars, would pave the path to next-generation microbial cell factories. However, opportunities for heterologous transporter engineering in thermophiles are bottlenecked by the limitations of reliable and high-throughput genetic manipulation; the current state of genetic systems for biotechnologically relevant thermophiles has been described in several reviews (Zeldes et al., 2015; Q. Yan & Fong, 2017; Freed et al., 2018; Straub et al., 2018; Riley & Guss, 2021; Lu et al., 2022). Utilizing biotechnologically relevant archaea and bacteria will also require separate advancements in archaeal and bacterial genetic tools due to lower genetic compatibility between species across these two domains of life (Q. Yan & Fong, 2017; Freed et al., 2018; Straub et al., 2018; Riley & Guss, 2021).

## Sugar Transport as a Metabolic Control Strategy

**Fig. 6.**
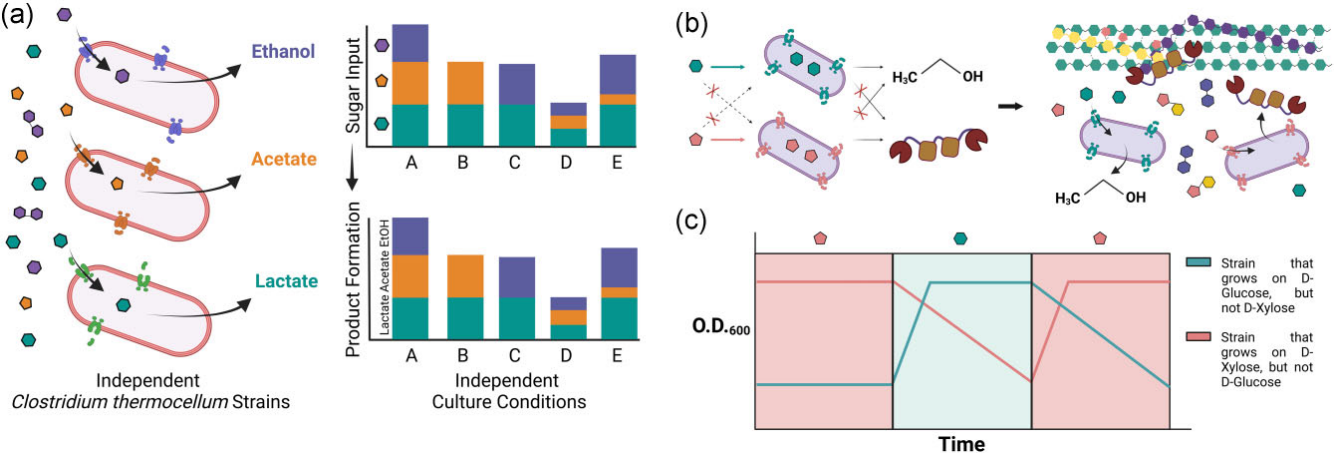
Sugar transport engineering at the cellular scale. (**a**) Sugar inputs proportionally control bioproduction outputs. (**b**) Construction of microbial strains with distinct sugar uptake patterns, enabling distribution of labor between a CAZyme-producing strain for lignocellulose deconstruction and a biofuel producing strain. (**c**) Integrating orthogonal sugar transport with cybergenetic modalities for dynamic control of co-culture composition. (Created using Biorender)

Cellular control of sugar transport can be leveraged for selective bioproduction. For example, *C. thermocellum* is capable of not only producing valuable biofuels like ethanol but also chemical commodities like acetate and lactate. By constructing independent strains with disabled ethanol, acetate, and lactate production, with each deficiency in product formation linked to disabled transport of specific sugars, we envision creating a synthetic consortium where the composition of sugar inputs would dictate the composition of product outputs (Fig. 6a). Here, strain composition is controlled by the proportion of sugars present, for example, increasing fructose input favors a fructose-consuming strain over a paired glucose-consuming strain.

Sugar transport also presents a new method for engineering microbial co-cultures. Microbial co-cultures benefit from modularizing complex expression pathways across multiple strains, reducing metabolic burden and the build-up of undesirable or toxic byproducts in any single cell, and increasing substrate utilization and biosynthetic efficiency (H. Zhang et al., 2015). Using *E. coli* as an example, Zhang et al. (2015) created a synthetic co-culture for the production of complex *cis, cis*-muconic acid and 4-hydroxybenzoic acid by distributing the pathway across xylose-utilizing and glucose-utilizing *E. coli* strains. Modularizing such biosynthetic pathways not only increased product yields but also simplified independent optimization of segments of the whole pathway. Combining a pentose-utilizing and hexose-utilizing strain for orthogonal sugar transport also circumvented carbon source competition.

This principle of orthogonal sugar transport is key to synthetic co-cultures comprising biomass degrading thermophiles. As microbial co-cultures of the same species exhibit greater stability and dynamics, the *Anaerocellum, Caldicellulosiruptor*, or *Clostridium* genera are especially promising metabolic engineering workhorses for this goal due to their broad sugar transporter inventory. And by demonstrating that orthogonal sugar uptake can be used to control co-culture growth dynamics, we can further engineer synthetic consortia to employ a division of labor, across individual strains, to enhance consolidated bioprocessing. For example, one strain could be selected for cellulase and hemicellulase over-production as the principal biomass degrader strain, while another strain is engineered with an inability to express CAZymes, shifting energy resources and metabolic flux toward bioproduction pathways as the producer strain (Fig. 6b). This distribution of labor is augmented by substrate cross-feeding and overcomes the challenges of population instability and poor control over strain composition (Fig. 6c; Chung et al., 2013; Sgobba & Wendisch, 2020; Duncker et al., 2021; Kang et al., 2022; T. A. Lee & Steel, 2022; X. Li et al., 2022). The initial inoculum ratio of selective sugar utilization strains as well as periodic pulsing of select carbon sources are especially facile tools with which to control metabolic product titers (H. Zhang et al., 2015). Pulsing of select carbon sources can also be interfaced with computer algorithms, expanding the toolkit of cybergenetic tools for control of microbial consortia (Fig. 6c). Outputs such as hydrogen gas or ethanol that are typical products of thermophilic fermentation can be used to autoregulate sugar inputs. That many lignocellulose degrading thermophiles possess minimum carbon catabolite repression also suggests the lower likelihood of one strain outcompeting the other during cross feeding, given similar utilization efficiencies on different carbon sources.

## Concluding Remarks and Future Perspectives

Over two decades of structural biology, biophysics, and transcriptomics have established the discovery and understanding of high-affinity sugar transport in thermophilic archaea and bacteria. Our abundant understanding of this phenomenon and emergent genome-editing tools in non-model thermophiles have reached the stage where sugar transport can be harnessed as a novel design paradigm in thermophilic synthetic biology.

Metabolic engineer David Fell remarked in “Understanding the Control of Metabolism” (1997) that “our ability to interfere with an organism's genetics has far outstripped our ability to predict the effects on its metabolism” (Fell, 1997). However, at the physiological extremes of synthetic biology, the reverse has tended to be the case. Sugar transporter engineering in thermophiles has historically been hamstrung by limited genetic tools. And so, like all great metabolic engineering strategies, progress in controlling sugar transport will depend on a tight interplay between a fundamental understanding of sugar uptake and building tools for process refinement. We envision whole-genome sequencing and transcriptomics to continue playing important roles in identifying both existing and new modes of sugar transport across thermophilic species; these discoveries in turn will rely on structural characterization and biophysical measurements to validate the identities of translocated sugars. Genome-editing technologies will need to be developed for those thermophiles without, while existing tools demand continuous optimization to reduce cycle times between genetic manipulations. The culmination of these efforts will be a robust synthetic biology strategy that transforms how thermophiles are utilized in industrial settings, and which can interface with contemporary metabolic engineering and bioprocessing technologies.

Realizing the future bioeconomy hinges on our ability to harness amongst the most recalcitrant of nature's carbon sources using microbial factories. Governing the assimilation of carbohydrates from lignocellulosic biomass for conversion into valuable fuels and chemicals, sugar transport marks an emerging yet strategically important area in synthetic biology and thermophile engineering.
